# Levels of Awareness and Concentrations of Heavy Metals in the Blood of Electronic Waste Scavengers in Nigeria

**DOI:** 10.5696/2156-9614-9.21.190311

**Published:** 2019-03-14

**Authors:** Oluseun E. Popoola, Abiodun O. Popoola, Diane Purchase

**Affiliations:** 1 Department of Chemical Science, Yaba College of Technology, Lagos, Nigeria; 2 Department of Radiology, Oncology Unit, Lagos State University, College of Medicine, Lagos, Nigeria; 3 Department of Natural Sciences, School of Health and Social Sciences, Middlesex University, London, UK

**Keywords:** electronic waste, scavengers, heavy metals in blood, dumpsite, awareness, Lagos

## Abstract

**Background.:**

Electronic waste (e-waste) contains both valuable and hazardous materials. E-waste scavengers specialize in the collection and crude recycling of waste electronics to retrieve valuable metals, which are then sold. These activities provide an income for scavengers, but also expose them to toxic heavy metals such as lead (Pb) and copper (Cu).

**Objectives.:**

The aim of the present study was to investigate the level of awareness and concentrations of heavy metals (Pb, Cu, zinc (Zn) and manganese (Mn)) in the blood levels of e-waste scavengers at Jakande dumpsite, Alaba International Market, Lagos, Nigeria.

**Methods.:**

Material and data were collected by empirical survey with the use of a questionnaire to obtain information from e-waste scavengers. Blood samples of the scavengers in the present study (30 adult males exposed to recycling processes) were collected and concentrations of heavy metals were determined through acid digestion and the use of an atomic absorption spectrophotometer (AGILENT 55B AA, 2010).

**Results.:**

The geometric means of blood levels of Pb, Cu, Zn and Mn were 11.0, 33.85, 126.15 and 19.38 μg / dL, respectively. High concentrations of Pb and Mn (11.0 and 19.38 μg / dL) were found in the blood samples, while Zn and Cu (126.15 and 33.85 μg / dL) showed low concentrations. The maximum blood level of lead (BPb) (24.0 μg / dL) was extremely high compared to the maximum BPb of occupationally exposed males. Statistical analysis of the questionnaires showed that all of the respondents were male, and more than half (56.7%) were between 21–30 years of age and had been involved in recycling of e-waste for 1–5 years. The results showed that 83% of the respondents were aware that hazardous fractions in e-waste require special treatment, while 76.7% were aware of the possible negative impact on their health.

**Conclusions.:**

Lack of education, poverty and lack of effective enforcement of e-waste management and regulations are the major contributors to the current situation and thus scavengers carry on with their activities unhindered. The authors recommend the use of protective clothing, sensitization visits and awareness campaigns on the safe disposal of hazardous components.

**Participant consent.:**

Obtained

**Ethics Approval.:**

The study was approved by the ethics committee of the Lagos State University Teaching Hospital, Ikeja Lagos.

**Competing Interests.:**

The authors declare no competing financial interests.

## Introduction

Electronic waste (e-waste) is defined as an unwanted electronics appliance (cell phone, music player, television, laptop, telecommunication equipment, etc.) that has reached the end of its useful life.^1^,^2^ This definition is relative, as an electronic product regarded as waste in a high-income country may become a resource in a low-income one.

The lifespan of computing and electronic goods is reducing at alarming rates in developed countries due to an increase in consumption and replacement. The need to dispose of or recycle the waste stream (waste electronics or e-waste) will rise as the number of obsolete items increases. Some of the e-waste that is deemed obsolete by developed nations is still of use in developing countries which accept e-waste items as second-hand equipment. These imported second-hand electronics serve as information and communication technologies for low-income earners who cannot afford new equipment.

Disposal and recycling of waste electronics in developed countries is more expensive and difficult due to stringent laws and regulations compared to poorer countries where enforcement is lacking. E-waste is thus exported to poorer countries where it may be landfilled or recycled using primitive techniques with little regard for worker safety or environmental protection.^3^ In countries where e-waste is regulated, producers are required to establish systems for collection and treatment of e-waste. However, in a previous study on e-waste recycling and disposal, up to 75% of items produced in the European Union and 80% in the United States go unaccounted for.^4^ According to Adaramodu et al., “over 80% of the world's e-waste ends up in landfills in Asia and Africa, and Nigeria is emerging as one of the top dumping grounds for toxic, chemical and electronic waste from developed countries”.^5^ It was reported that an average of 500 containers containing used electronics are imported into Lagos Port each month. According to the Computer and Allied Product Dealers Association of Nigeria, about 75% of those electronics shipped into the country are irreparable waste.^6^ The remaining 25%, some of which are still functional (for re-use or refurbishing), are in the second stage of their life span (i.e. passive life) and would soon be discarded. This trend is adding to the growth of e-waste in Nigeria.

Electronic devices form a complex mixture of materials and components often containing several hundreds of different substances, many of which are toxic and create serious pollution upon disposal. These include heavy metals such as mercury (Hg), lead (Pb), cadmium (Cd), chromium (Cr) etc., organic substances such as flame retardants (polybrominated biphenyls, polybrominated diphenylethers and others, including polyaromatic hydrocarbons, polychlorinated biphenyls, phthalates, etc.). E-waste contains both valuable and hazardous materials that require special handling and recycling methods to avoid environmental contamination and detrimental effects to human health.^7^ Recycling can recover reusable components and base materials, especially copper (Cu) and precious metals.

**Abbreviations**
*BCu*: Blood copper level
*BLL*: Blood lead level
*BMn*: Blood manganese level
*BZn*: Blood zinc level

Waste collectors/scavengers collect waste from the street. Some scavengers specialize in the collection of electronic wastes. They dismantle e-waste manually and retrieve valuable components such as gold, metal, copper, etc. These materials are then sold as a means to earn a living. Scavenging is considered a lucrative business, yet the health effects connected to the exposure of toxic substances is under-acknowledged. Scavengers engage in dismantling (mostly with screw drivers and bare hands) and burning e-waste to retrieve valuable metals and other materials, and through this process they are exposed to harmful chemicals.^8^ Inhalation of fumes, direct contact and dust ingestion are the most prominent routes of human exposure to hazardous substances and may lead to the accumulation of toxic metals in the body which may pose serious health risks.^9^ Uncontrolled disposal and crude recycling of e-waste has contributed immensely to the pollution of the environment. The existence of this informal recycling sector has often led to careless handling of e-waste and a lack of knowledge of the dangers involved.^10^,^11^ It is common to see scavengers rummaging through solid waste heaps at dumpsites without concern for possible health implications.

According to Adediran and Abdulkarim, there has not been any serious initiative to deal with the management of e-waste in Nigeria.^12^ Although institutional frameworks such as that of the National Environmental Standards and Regulations Enforcement Agency are in place, their efforts are yet to be fully effective.

The current elements of e-waste management in Nigeria are as follows:

- There is no legislation to control the flow of used consumer electronic products.
- The Nigeria Customs Service does not regard used electronic products as contraband as long as the appropriate duties and taxes are collected on them.
- There is no public awareness of the inherent dangers of handling e-waste, which is regarded as a business opportunity.
- There are no e-waste recycling facilities in the country.
- There is no corporate social responsibility on the part of industries for e-waste.^12^

This investigation aims to explain the effects of improper recycling methods, such as burning, on the health status of e-waste scavengers. It is a pilot study to obtain preliminary data in order to establish whether recruitment for future study is feasible in this hard to reach population (e-waste scavengers) as well as to determine if blood heavy metal levels are concerning. It is hoped that the information/data provided will advocate for more stringent governmental policies on the importation and recycling of e-waste in a low- and middle-income country such as Nigeria.

## Methods

**Figure 1:**
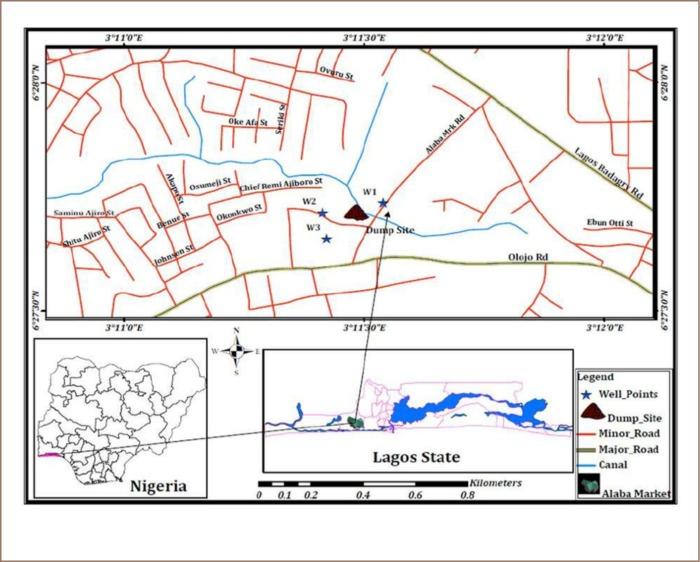
Jakande e-waste dumpsite in Alaba International Market in Lagos State

The present study was carried out at the Jakande e-waste dumpsite in Alaba International Market in Lagos State (*Figure 1*). Lagos State is located in southwestern Nigeria at latitude 6°28′ N and longitude 3°10′ E. The tropical climate has a wet season from April–October and a dry season from October–May. Based on the 2006 census, Nigeria has a population of 152,217,341 with a growth rate of 1.9%, birth rate of 36.0/1000, infant mortality rate of 92.9/1000, and life expectancy of 50.2 years.^13^

### Sampling

A total of 40 male, adult subjects between 21 and 60 years of age volunteered to participate in the present study. The study was unable to identify any female scavengers. The job of E-waste scavenging is dominated by males from northern Nigeria who are primarily Muslims. Scavengers perform their work without safety precautions, dismantling and crudely recycling e-waste with open fire burning. Sensitization visits were carried out prior to the start of the study. Questionnaires were distributed on site, while blood sample collection was preformed at a nearby, designated location. Forty questionnaires were distributed, however only 30 were properly filled out and returned. The biggest barrier to filling out the questionnaires properly was a language barrier, as many scavengers were from northern Nigeria and spoke only Hausa, with little or no understanding of English. Informed consent was obtained from respondents, and they were educated on the benefits of the study, as many were unaware of the negative effects of scavenging activities. Crude recycling activities have taken place in this area for many years, and several tons of computers and electronics waste are handled each year.^14^

The convenience sampling technique was used to select samples from the population for the present study. A total of 30 questionnaires were administered to the target audience, 23 e-waste scavengers and 7 controls. The refusal rate was generally high. Inclusion criteria consisted of adults between 21–60 years old involved in e-waste scavenging. Exclusion criteria included individuals with previous experience in metal mining. Individuals with previous metal mining experience were excluded because this experience could be responsible for high blood metal levels.

### Research instrument

The study survey focused on the level of awareness of government regulation, adherence to e-waste management, as well as concern about the environment. It also investigated the respective respondents' mode of disposal of e-waste as well as their awareness of the potential harm of e-waste. The questionnaires were distributed by hand, which allowed for individual interaction with the respondents. The study questionnaire is included as Supplemental Material.

### Data analysis methods

Inferential statistics were used for the data analysis in the present study, as sampling naturally incurs sampling errors and the sample does not perfectly represent the population.

Blood samples were obtained from scavengers working at the Jakande dumpsite with the assistance of medical practitioners. Ethical approval was obtained from the Health Research and Ethics Committee, Lagos State University, Teaching Hospital, Ikeja, Lagos. The blood was preserved in heparinized bottles and stored in a refrigerator. Disposable pyrogen-free needles and syringes were used to withdraw 10 ml of blood from the antecubital vein. The blood samples were dispensed into plain vacutainer tubes containing ethylenediaminetetraacetic acid to prevent coagulation. Samples were kept frozen at −40°C until analysis.

### Digestion of blood samples and determination of heavy metals

Blood samples were retrieved from the freezer and allowed to thaw. Then 1 ml of the blood sample was drawn into a conical flask. Afterwards, 10 ml of concentrated HNO_3_ was added to the flask and subsequently heated on a hot plate. When the fumes became clear and the solution was nearly colorless, the solution was removed and allowed to cool. Then, the solution was brought to 25 ml by adding de-ionized water and stirring.^15^ The digested samples were analyzed using an atomic absorption spectrophotometer (Alpha 4 (AGILENT 5AA)) to determine heavy metal (Pb, Zn, manganese (Mn) and Cu) concentrations in the blood samples.

## Results

A summary of results from the questionnaire at Jakande waste dumpsite in Alaba International Market is shown in Table 1. Results of the awareness of respondents of the activities of e-waste scavengers are presented in Table 2.

**Table 1:** Demographics of Questionnaire Respondents in Jakande Waste Dumpsite in Alaba International Market (N=30). ^*^Fishers exact test significant at P<0.05

**Age group (years)**			
21–30	15 (65.2)	2 (28.6)	17 (56.7)
31–40	6 (26.1)	4 (57.1)	10 (33.3)
41–50	2 (8.7)	0 (0.0)	2 (6.7)
>51	0 (0.0)	1 (14.3)	1 (3.3)

**Marital status**			
Single	15 (65.2)	1 (14.3)	16 (53.3)
Married	8 (34.8)	6 (85.7)	14 (46.7)

**Working experience (years)**			
1–5	9 (39.1)	2 (28.6)	11 (36.7)
6–10	5 (21.7)	3 (42.9)	8 (26.7)
11–15	6 (26.1)	1 (14.3)	7 (23.3)
16–20	3 (13.0)	1 (14.3)	4 (13.3)

**Level of education**			

None	6 (26.1)	0 (0.0)	6 (20.0)

Primary	7 (30.4)	2 (28.6)	9 (30.0)

Secondary	8 (34.8)	3 (42.9)	11 (36.7)

Technical	0 (0.0)	1 (14.3)	1 (3.3)

National Certificate of Educati(NCE)on	1 (4.4)	1 (14.3)	2 (6.7)

Ordinary National Diploma	1 (4.4)	0 (0.0)	1 (3.3)

College	-	-	-

**Trade association membership**			

No	6 (26.1)	2 (28.6)	8 (26.7)

Yes	17 (73.9)	5 (71.4)	22 (73.3)

**Table 2:** Awareness of Respondents of the Activities of E-waste Scavengers (N=30)

**Variables**	**Scavengers N (%)**	**Non Scavengers (%)**	**Total N (%)**
**Awareness of governmental regulation of e-waste management**			
No Yes	9 (39.1) 14 (60.9)	4 (57.1) 3(42.9)	13 (43.3) 17 (56.7)
**Concern about the environment**			
Not concerned Somewhat concerned Concerned Very Concerned	2 (8.7) 2 (8.7) 9 (39.1) 10 (43.5)	3 (42.9) 1 (14.3) 0 (0.0) 3 (42.9)	5 (16.7) 3 (10.0) 9 (30.0) 13 (43.3)
**Awareness of toxic and hazardous components of electronic devices**			
No Yes	5 (21.7) 18 (78.3)	1 (14.3) 6 (85.7)	6 (20.0) 24 (80.0)
**Awareness of the impact of disposal/treatment method on the environment**	2 (8.7) 18 (78.3) 3 (13.0)	1 (14.3) 6 (85.7) 0 (0.0)	3 (10.0) 24 (80.0) 3 (10.0)
No Yes Not sure			
**Awareness of the negative impact on health from handling or being in contact with electronic waste**	2 (8.7) 17 (73.9) 4 (17.4)	1 (14.3) 6 (85.7) 0 (0.0)	3 (10.0) 23 (76.7) 4 (13.3)
No Yes Do not know			
**Awareness of the recovery of electronic equipment/components from waste**	0 (0.0) 23 (100.0)	1 (14.3) 6 (85.7)	1 (3.3) 29 (96.7)
No Yes			

### Questionnaire analysis

Table 1 presents responses to the questionnaire survey. There was a significant association between marital status and profession. Tables 3 and 4 indicate respondents' awareness of e-waste collection hazards and the environmental sectors they consider to be most affected by e-waste. Tables 5 show the nature and ranking of common health hazards that scavengers reported experiencing, while Table 6 show the blood concentrations of heavy metals in scavengers in the present study compared to other studies. Most of the scavengers were secondary/primary school dropouts with no formal education. With regard to whether waste management rules are effectively enforced, 50% of scavengers strongly disagreed, 43% reported some disagreement and 7% of the scavengers stated that the rules were enforced, as shown in Table 3.

**Table 3:** Scavenger Awareness of E-waste Management Issues

	Frequency	Percentage
**Awareness of the enforcement of waste management rules**		
Yes	2	7%
No	15	50%
Somewhat	13	43%
**Awareness that the components in electronic devices require special treatment for environmentally sound disposal**		
Yes	21	70%
No	9	30%
**Awareness that some hazardous fractions in electronic waste need special treatment**		
Yes	25	83%
No	5	17%

**Table 4:** Environmental Sectors Most Affected by the Activities of E-waste Scavengers as Indicated by Respondents

**Affected environmental sector**	**Very Strong**		**Strong**		**Fair**		**No effect**		**Mean**	**Rank**
	**Freq.**	**%**	**Freq.**	**%**	**Freq.**	**%**	**Freq.**	**%**		
Land	25	83.3	2	6.7	2	6.7	1	3.3	3.7	2nd
Air	25	83.3	4	13.3	1	3.3	0	0.0	3.8	1st (most affected)
Surface water	19	63.3	6	20.0	1	3.3	4	13.3	3.3	3rd
Underground water	18	60.0	8	26.7	0	0.0	4	13.3	3.3	3rd
Vegetation	16	53.3	5	16.7	5	16.7	4	13.3	3.1	5th (least affected)

**Table 5:** Health Effects Reported by Scavengers

**Health hazard**	**Very Severe**		**Severe**		**Fair**		**Not Severe**		**Mean**	**Rank**
	**Freq.**	**%**	**Freq.**	**%**	**Freq.**	**%**	**Freq.**	**%**		
Head ache	12	40.0	3	10.0	9	30.0	1	3.3	2.53	3rd
Body pain	17	56.7	2	6.7	2	6.7	3	1.0	2.70	1st (most reported)
Dizziness	10	33.3	7	23.3	3	10.0	2	6.7	2.30	4th
Cough	14	46.7	4	13.3	2	6.7	5	16.7	2.57	2nd
Other (catarrh, heart pain, cold, stomach, chest pain, heart burn, weakness, heart disorder)	9	30.0	0	0.0	0	0.0	1	3.3	1.23	5th (least reported)

**Table 6:** Concentrations of Heavy Metals (μg/dL) in the Blood of E-Waste Scavengers in Jakande Waste Dumpsite in Alaba International Market, Lagos Compared with Similar Studies in Nigeria

**Sample ID**	**Pb (μg/dL)**	**Zn (μg/dL)**	**Cu (μg/dL)**	**Mn (μg/dL)**	**Study**
Blood Sample 1	2.0	120	40	10	Present study
Blood Sample 2	22.0	190	30	20	Present study
Blood Sample 3	24.0	160	40	40	Present study
Blood Sample 4	21.0	100	20	10	Present study
Blood Sample 5	11.0	70	20	10	Present study
Blood Sample 6	4.0	190	30	30	Present study
Blood Sample 7	16.0	70	100	10	Present study
Blood Sample 8	11.0	60	20	20	Present study
Blood Sample 9	<1.0	120	30	10	Present study
Blood Sample 10	4.0	40	30	20	Present study
Blood Sample 11	16.0	190	40	40	Present study
Blood Sample 12	8.0	220	20	30	Present study
Blood Sample 13	3.0	110	20	2	Present Study
Mean of metals in Blood	11.0	126.15	33.85	19.38	Present study
^*^Control (non-exposed male)	30.0 μg/dL	368.0 μg/dL	32	-	Present study
Blood Levels (18–45 yr, exposed male auto mechanics)	27.00 to 48.90				Abeokuta, Southwest Nigeria^16^
Blood Levels (18–45 yr, exposed male paint factory workers)	39.00 μg/dL				Nkpor, Southeast, Nigeria^17^
Blood Levels (18–45 yr, non-exposed, male control subjects)	15.78 μg/dL				Abeokuta, Southwest Nigeria^16^
Blood Levels (18–45 yr, non-exposed, male control subjects)	17.00 μg/dL				Nkpor, Southeast, Nigeria^17^

^*^Individuals that are not occupationally exposed to heavy metals such as students

As shown in Table 2, 80% of the respondents were aware of the adverse effects of the crude dismantling of e-waste. Seventy percent (70%) were aware that these toxic/hazardous materials require special treatment for environmentally sound disposal and 80% were aware of the impact of disposal/treatment method on the environment. Scavengers felt that air was the most highly impacted by e-waste, followed by land, surface and underground water (tied) and vegetation. In addition, 76.7% were aware of negative health impacts such as body pain, cough, headache, dizziness, etc.

Techniques of disposal of electronic wastes in Nigeria include sale to repair shops (63.3%), recyclers (63.3%) and reuse (50%). In the present study, 83% of scavengers were aware that some hazardous fractions in electronic waste need special treatment, as shown in Table 3. Generally, scavengers dismantle electronic equipment manually using a hammer, chisel, saw, or by hand. Scraps of electronic equipment that have no further value are disposed of indiscriminately.

## Discussion

The present study investigated e-waste scavengers' exposure to heavy metals, identifying their daily exposure to crude e-waste recycling as a major occupational hazard. Most scavengers refused to participate voluntarily due to the illegal status of their operations. They felt that their participation in the study would have negative consequences, which is reflected in the small sample size of respondents. Low participation could also be attributed to cultural belief in this part of the world that the blood samples might be used for ritual purposes. Thus, the major limitation of the present study was its very small sample size. The preliminary data obtained in this pilot study indicates that recruitment is feasible in this hard to reach population. Therefore, further investigation with a larger sample size over a longer time frame should be possible. With regard to whether waste management rules are effectively enforced, 50% strongly disagreed, 43% reported some disagreement and 7% of the scavengers stated that the rules were enforced. This is in accordance with the paper by Adediran and Abdulkarim, 2012, who reported little or no effective enforcement of regulations related to e-waste management and disposal in Africa.^12^,^15^ A study by Oteng-Ababio in 2013 revealed that e-waste contains valuable metals like copper, gold and silver.^29^ In the face of high unemployment and poverty, scavengers engage in crude recycling because it provides an income. A study by Owusu-Sekyere in 2014 also found that most scavengers have their current occupation not by choice, but due to high levels of unemployment and poverty.^30^

The concentrations of heavy metals in the blood of e-waste scavengers in Jakande e-waste dumpsite, Alaba International Market, Ojo, Lagos were determined. The high blood level of lead (BLL) and blood level of manganese (BMn) of the scavengers is concerning, there is thus need for further studies. The geometric mean of BMn in the present study was 19.4 μg/dL (*Table 6*), and these values exceed the suggested normal range of 8.01 μg/L- 9.98 μg/L in an Italian population as reported by Bocca et al.^26^ They are also higher than levels reported in other countries, as shown in Table 8. E-waste scavengers have high levels of Mn in their blood and is therefore possible for them to be affected by neuro-generative disorders and diseases as observed in a study by Crossgrove and Zheng.^31^ Manganese is an essential element in the human diet which serves many cellular functions in the human body, however elevated BMn levels can result in a Parkinson's disease-like syndrome. However, blood levels of Mn are not usually reliable indicators of toxicity because they are quickly metabolized in the body. The concentrations in the present study indicate that continuous exposure subjects these workers to high-risk neurological disorders.^32^

**Table 7:** Geometric Mean of Heavy Metals Concentration in the Blood of E-Waste Scavengers

**Heavy metals (Ug/dL)**	**Mean (μg/dL)**	**Standard deviation**	**95% Confidence Interval**
**Lead**	110.0	81.2	60.91–159.09
**Zinc**	126.2	58.7	90.69–161.60
**Copper**	33.9	21.4	20.89–46.79
**Manganese**	19.4	12.3	11.94–26.82

**Table 8:** Mean Metal (Mn, Cu, Zn and Pb, μg/dL) Concentrations in Blood of E-Waste Scavengers in Lagos, Nigeria Compared with the Blood of Non-exposed Populations^*^ in Other Countries

	**Mean blood level of manganese**	**Mean blood level of copper**	**Mean blood level of zinc**	**Mean blood level of lead**
**China^18^**	1.14	80.24	466.5	4.26
**America^19^**	0.4–1.5			1.23
**Australia^20^**				1.45
**Brazil^21^**	0.96	89		6.54
**Canada^22^**	1.08			2.13
**Czech^23^**		80	580	3.31
**Denmark^24^**	0.91			4.83
**Germany^25^**	0.86	102		1.93
**Italy^26^**	0.89	103.6	641.8	3.34
**Korea^27^**	1.08			1.94
**Spain^28^**		107	695	0.1
**Present Study**	19.4	33.85	126.2	11.0

Copper is an essential nutrient that is required for numerous metalloenzymes such as tyrosinase, cytochrome-oxidase, superoxide-dismutase, etc.^33^ The mean blood level of copper (BCu) in the present study was 33.85 μg/dL, which is low compared to results of the Chinese national survey (802.0 μg/dL), and considerably lower than the values reported in other studies.^18^ Copper is an essential element in the body and the low levels of Cu in the blood of scavengers in this study make them susceptible to the risk of developing coronary heart disease, anemia, etc.^34^ Manganese and Cu are essential to human health, but excessive exposures may have adverse health effects.^35^

Zinc plays a pivotal role in human health, it is as important in enzymes that are essential for intracellular processes and deficiencies cause major clinical conditions.^36^ By conservative estimates, nearly 25% of the world population is at risk of Zn deficiency.^37^ The mean blood level of zinc (BZn) in the present study (126.15 μg/dL) suggests that BZn levels are very low compared to the Chinese national survey value (4665 μg/dL) and also considerably lower than in other countries (*Table 8*).^18^ The low levels of BZn found in the scavengers' blood could result in growth retardation, anorexia, delayed sexual maturation, mental retardation and impaired immunological function.^37^

Lead is very toxic and can induce various cancers and diseases.^38^ The United States Environmental Protection Agency categorizes Pb compounds as carcinogens, neurotoxicants and neuro-developmental toxicants.^38^ Oxidative stress caused by reactive oxygen species is a well-known mechanism of heavy metal-induced damage. BLL levels in the present study were very high (11.0 μg/dL) compared with the Chinese national survey value (4.26 μg/dL) and the reference ranges of other countries (*Table 8*).^18^The present study links high BLLs in e-waste scavengers to their constant exposure to e-waste toxins through open fire burning, cutting, shredding and direct inhalation of smoke coming from the burning e-waste materials. Lead is toxic to the human body at any level, but manageable at very minimal levels. The maximum BLL (24.0 μg/dL) was lower compared to the maximum BLL of other occupationally exposed males.^39^ The BLL concentrations in traffic wardens and police in different parts of Lagos were found to be 152.42 μg/dL in Oshodi, 148.56 μg/dL in Dopemu and 122.6 μg/dL at the Ojota bus stop, reflecting different levels of traffic congestions across the city of Lagos.^40^

One limitation of the present study was the small number of questionnaire respondents and blood samples. The poor electricity supply in Nigeria was a problem, as some blood samples were hemolyzed and thus could not be analyzed. The absence of female scavengers is also a major limitation of the study. It is possible that the cultural and religious beliefs of Muslims do not encourage the participation of women in any vocation dominated by males. The few women and children encountered at the site of study were there to sell food and drinks to the scavengers. Further studies are needed with a larger and more diverse sample group.

## Conclusions

The results of the present study provide information on the concentration of heavy metals (Pb, Mn, Zn and Cu) in the blood of e-waste scavengers in Jakande e-waste dumpsite, Alaba International Market, Lagos. The results indicate that scavengers are aware of the effects of crude recycling, improper disposal of e-waste and its negative impact on the environment and their health. However, due to economic challenges and high unemployment, scavengers have few occupational alternatives.

The data in the present study can help to establish reference values for metals levels in the blood of scavengers. BLL and BMn concentrations were very high, but the levels of BCu and the BZn were low compared to the normal ranges. High BLL and BMn can cause health problems to exposed individuals later in life. Additionally, the very low levels of BZn and BCu of scavengers indicate deficiencies which may have deleterious health consequences.

### Recommendations

The preliminary data indicate that recruitment is feasible in this hard to reach population and further studies are recommended. The high BLL and BMn in scavengers is concerning and continuous monitoring is needed. The government should ban crude e-waste recycling activities and engage scavengers in some other vocation, enforce laws on the use of safety equipment by waste scavengers, even in the informal e-waste recycling sector, and build modern e-waste recycling facilities. The creation of a specialized center managed by a multidisciplinary team of experts composed of physicians, toxicologists, chemists, politicians, environmentalists and social workers could help to address metal pollution in Nigeria and the needs of individuals with a BLL > 20 μg/dL. Finally, the government should strongly restrict the importation of second-hand electronics and substandard products from developed countries.
