# Supplementary material for: Levels of Awareness and Concentrations of Heavy Metals in the Blood of Electronic Waste Scavengers in Nigeria
Source: J Health Pollut. 2019 Mar 14;9(21):190311. doi: 10.5696/2156-9614-9.21.190311 (PMC6421953; doi:10.5696/2156-9614-9.21.190311)
Supplement: Supplementary file 1 [file hapn-9-21-190311_s01.docx]

**Supplemental Material**

**Questionnaire on Environmental and Health Effects
of Electronic Waste Recycling**

**Introduction:**

Dear respondent,

We are pleased to invite you to participate in this research project intended to determine the **Individual Awareness and Heavy Metals in Blood of Scavengers Involved in the Indiscriminate Recycling of Electronic Waste** with the hope that the study findings may enable a more holistic approach to the management and disposal of electronic waste in Nigeria and other countries. This will help to determine the extent of your exposure to these toxic contaminants (heavy metals) in e-waste and their effects on the environment. All information collected from you will be treated with complete confidentiality. Your participation is voluntary and if you decline participation, this will not affect you in any way.

WORKER INFORMATION

1. Age:

2. Marital status: ________________________

3. Any formal education? Specify the level of education

4. How long have you been involved in scavenging?

QUESTIONS ON AWARENESS AND BEHAVIOR

5. Do you belong to any workers’ association? Yes / No

6. If yes, name the association and its address:

7. Are you aware of any governmental regulation on e- waste management? Yes /No

8. Do you have rules guiding your activities in the association? Yes / No

9. If yes, are the rules in compliance with government regulation for waste
management?

Yes /No. Others (please specify)

10. How effectively are the rules enforced? Strongly / Not strongly / Not enforced

11. Are you concerned about your environment? Not concerned / Barely concerned / Concerned / Very concerned.

12. Do you know that some components of electronic waste contain toxic/hazardous
materials?

Yes / No

13. If yes, are you aware that these toxic/hazardous materials require special treatment for environmentally sound disposal? Yes / No

14. Does the disposal treatment method in use for electronic waste have any impact on the environment? Yes / No / Not sure

15. If yes, indicate the part(s) of the environment affected and rate the impact

IMPACT

PARTS OF ENVIRONMENT VERY STRONG STRONG FAIR NO EFFECT

AFFECTED

Land

Air

Surface water

Underground water

Vegetation

16. Does your involvement in handling of/ being in contact with electronic waste have a
negative impact on your health? Yes / No / Do not know.

17. If yes, what is the nature of the health hazard? Rate severity.

18. Severity

NATURE OF HEALTH Highly severe Severe Fair Not severe

Headache

Body pain

Dizziness

Cough

Others specify

20. What volume of electrical/electronic waste do you handle per day?

21. Do you recover any of the electronic equipment/components from waste? Yes / No

22. If yes, which equipment? PC / Laptop / TV / Mobile phone

23. What kind of materials do you extract from these electronics?

- Glass…………………….Yes / No
- Battery………………….Yes / No
- Copper wires…………Yes / No
- Plastics…………………..Yes / No

24. What method do you use to dismantle the electronic equipment?

25. What do you do with the recovered equipment/components? Sell to repairers / Sell to recyclers / Reuse

26. From where and from whom do you collect unused electronics?

27. Are the electronics that you collect purely waste? Yes / No

28. What method do you use to dismantle and recycle e-waste?

29. Are you aware that some hazardous fractions in e-waste need special treatment in

order to be safely disposed of? Yes / No

30. Are you aware that some hazardous fractions in e-waste need special treatment in

order to be safely disposed of?

31. How do you dispose of scraps from the electronic equipment that is of no use?

32. Why do you recycle?

33. What volume/quantity of electronic equipment do you process per day, week, month or year?

34. How much income is generated from selling the extracted materials? (monthly or

annually).

35. Do you enjoy what you do?

If yes, why?

If no, why?
